# High degree of kinesiophobia after lumbar disc herniation surgery: A cross-sectional study of 84 patients

**DOI:** 10.3109/17453674.2011.636674

**Published:** 2011-11-25

**Authors:** Gunilla Limbäck Svensson, Mari Lundberg, Hans Christian Östgaard, Gunilla Kjellby Wendt

**Affiliations:** ^1^Department of Orthopaedics, Institute of Clinical Sciences, Sahlgrenska Academy, University of Gothenburg; ^2^Department of Physiotherapy and Occupational Therapy, Sahlgrenska University Hospital, Gothenburg; ^3^Department of Clinical Neuroscience and Rehabilitation/Physiotherapy, Institute of Neuroscience and Physiology, Sahlgrenska Academy, University of Gothenburg, Sweden

## Abstract

**Background and purpose:**

Several studies have investigated outcomes after disc surgery. However, the occurrence of kinesiophobia has not been investigated previously in patients after disc herniation surgery. In this cross-sectional study, we investigated kinesiophobia in patients who had been treated surgically for lumbar disc herniation, and we related the results to established outcome measures.

**Patients and methods:**

10–34 months after surgery, questionnaires were sent to 97 patients who had undergone standardized open discectomy. Outcome measures included Tampa scale for kinesiophobia (TSK); Oswestry disability index (ODI); European quality of life in 5 dimensions (EQ-5D); visual analog scale (VAS) for leg and back pain, work disability, and patient satisfaction; Zung self-rating depression scale (ZDS); pain catastrophizing scale (PCS); and a self-efficacy scale (SES).

**Results:**

36 of 80 patients reported having kinesiophobia. There were statistically significant differences in ODI, EQ-5D, VAS leg and back pain, ZDS, PCS, and SES between patients with and without kinesiophobia.

**Interpretation:**

Half of the patients suffered from kinesiophobia 10–34 months after surgery for disc herniation. These patients were more disabled, had more pain, more catastrophizing thoughts, more symptoms of depression, lower self-efficacy, and poorer health-related quality of life than patients without kinesiophobia.

Numerous studies have investigated outcomes after disc surgery and found poor results in 10–35% of the patients, depending on what outcome measure was used (Loupasis et al. 1999, Gotfryd and Avanzi 2009, Lundin et al. 2009). In a summary of outcome assessments for treatment of spinal disorders, the following domains were recommended to be included: back-specific function, generic health status, pain, work disability, and patient satisfaction (Bombardier 2000). Results from a systematic review indicated that socio-demographic, clinical, work-related, and psychological factors predict outcome of lumbar surgery outcome (den Boer et al. 2006).

In addition, affective factors, particularly fear, have proven to be central in explaining and in understanding of persistent musculoskeletal pain. 3 terms are used to describe fear in relation to pain: pain-related fear, fear of movement, and kinesiophobia. Pain-related fear is a broad and general term that covers all kinds of fears related to pain (Crombez et al. 1999). Fear of movement/(re)injury is described as “a specific fear of movement and physical activity that is (wrongfully) assumed to cause reinjury” (Vlaeyen et al. 1995a). In the most extreme situation of fear of movement, the expression “kinesiophobia” is used (Kori et al. 1990).

Kinesiophobia is considered to play a negative role in the outcome of rehabilitation for patients with low back pain, and a high prevalence of kinesiophobia has been observed in patients with persistent low back pain (Picavet et al. 2002, Lundberg et al. 2004). Since physical activity/exercise is a crucial part of the rehabilitation program after surgery, kinesiophobia is probably a factor that prevents recovery. However, a subgroup analysis of kinesiophobia has not been investigated previously in patients who have undergone disc herniation surgery. We studied kinesiophobia in patients who were treated surgically for lumbar disc herniation, and related the results to established outcome measures.

## Patients and methods

The present study had a cross-sectional design. The regional ethics review board approved the study (number Ö 246-03). All 97 patients between 18 and 65 years of age who had undergone standardized open discectomy during 2004 and 2005 in Södra Älvsborgs Hospital (Sweden) were invited to participate in the study. Discectomy was offered to patients with severe leg pain that had not resolved after at least 6 weeks, if CT or MRI showed disc herniation that would explain the clinical findings. After surgery, the patients were recommended to exercise according to an early, active rehabilitation program (Kjellby-Wendt et al. 2001). Questionnaires were sent to the patients in September 2006. If no response was received after 2 mailed reminders, the patients were reminded by telephone. 84 (48 men) of 97 patients returned the questionnaires (Figure). The patients had a mean age of 43 (11) years (Table 1).

**Figure:**
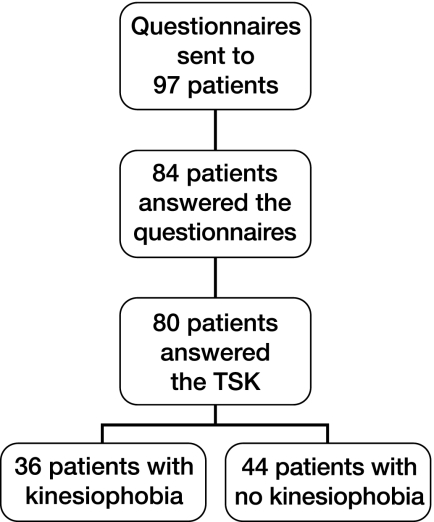
Exclusion and enrollment of participants.

**Table 1.** Descriptive data for the 84 patients who answered the questionnaires, 80 of whom answered the TSK, and those patients were classified as having kinesiophobia or no kinesiophobia

Characteristics	Returned the questionnaires	Kinesiophobia **^a^**	
		Yes	No
	n = 84	n = 36	n = 44
Men	48	22	23
Age, mean (SD)	43 (11)	42 (11)	43 (10)
Co-morbidity	26	12	12
Duration of symptoms preoperatively			
< 3 months	9	7	2
3–12 months	28	9	17
2 years	15	7	7
> 2 years	32	13	18
Surgery: > 1 operation	20	9	10
Disc herniation level:			
L2–L3	1	1	0
L3–L4	5	4	1
L4–L5	30	12	17
L5–S1	46	19	25
L4–L5 & L5–S1	2	0	1

**^a^** Kinesiophobia is defined as > 37 on the Tampa scale for kinesiophobia (TSK). 80 patients answered the TSK

### Outcome measures

The descriptive data included age, sex, duration of pain before surgery, and history of previous disc herniation surgery.

### Kinesiophobia

The Tampa scale for kinesiophobia (TSK) questionnaire comprises 17 items scoring the subjective rating of kinesiophobia. The score varies between 17 and 68, and Vlaeyen et al. (1995b) defined a cut-off of > 37 as showing a high degree of kinesiophobia. The TSK-SV has been found to be reliable and valid for use in Swedish patients with persistent low back pain (Lundberg et al. 2004).

### Back-specific function

The Oswestry disability index (ODI) comprises 10 items assessing back-specific function (Fairbank et al. 1980). The total score is expressed as a percentage, where 0% represents no disability. An ODI disability score of 0–20% is defined as minimal or no disability and a score > 40 is defined as severe disability (Fairbank et al. 1980).

### Health-related quality of life

The European quality of life in 5 dimensions questionnaire (EQ-5D) was used for measurement of health-related quality of life (HRQoL) (Rabin and de Charro 2001). The EQ-5D has 2 parts: EQ-5D^index^ and EQ-5D^VAS^. EQ-5D^index^ ranges from –0.59 to 1.0 where 1.0 is optimal health. The mean EQ-5D^index^ is 0.86 for a Swedish population aged 40–49 years old (Burström et al. 2001); therefore, a value of ≥ 0.86 could be defined as normal for this age group. The second part (EQ-5D^VAS^) ranges from 0 (worst possible health state) to 100 (best possible health state).

### Pain

Pain intensity was rated on 2 visual analog scales (VAS), 1 for leg pain and 1 for back pain (Scott and Huskisson 1976). The VAS ranges from 0 to 100 mm representing “no pain” to “maximal pain”. A score of 0–10 mm is defined as no pain (Öberg et al. 2002).

### Work disability

Work status was measured using a 3-grade Likert scale: working full-time, full-time sick leave, and part-time sick leave.

### Patient satisfaction

Patient satisfaction with treatment was measured on a 3-grade Likert scale: satisfied, less satisfied, and dissatisfied (Strömqvist et al. 2001).

### Depressive symptoms

The Zung self-rating depression scale (ZDS) scores depressive features on a scale from 20 to 80 (Zung 1965). The more depressed the patient is, the higher the score obtained. A score of 35 or higher indicates depressive symptoms (Zung 1965, Arpino et al. 2004).

### Catastrophizing thoughts

The Pain catastrophizing scale (PCS) scores catastrophizing thoughts on a scale from 0 to 52 (Sullivan et al. 1995). Patients scoring above 24 on the PCS are classified as catastrophizers and those scoring below 15 are classified as non-catastrophizers (Sullivan et al. 1995).

### Self-efficacy

The self-efficacy scale (SES) assesses functional self-efficacy beliefs specifically related to various basic physical activities (Estlander et al. 1994). The total range of score is 8–64, with higher scores indicating more positive beliefs.

### Statistics

Results are presented as median values and range, except for age, which is presented in mean (SD). Statistical comparisons between those with and without kinesiophobia were made from logistic regression with adjustment for age and sex. Comparison without adjustment was calculated with the chi-square test, with pooling of categories when necessary. The significance level was set at 5%. Statistical analyses were carried out using SPSS software version 18.

For 5 patients, 1 TSK item was missing and we used imputation with linear regression to replace the lost information. The imputation technique used may lead to an underestimation of the variance, but the small number of imputed data made this a minor problem. The ODI score was calculated as the sum of the ODI items divided by the number of valid items. In 4 patients, 1 item was missing.

## Results

26 of 84 patients reported co-morbidity involving a variety of different diseases and illnesses (Table 1). 13 patients declined to answer the questionnaires—9 of whom were men; mean age was 41 (9). 20 of 84 patients had been operated earlier for lumbar disc herniation (Table 1). Of those 20, 18 were had been operated twice, 1 patient had been operated 3 times, and 1 patient had been operated 5 times. Of the patients who declined to answer the questionnaires, 2 patients had been operated earlier. The distributions of disc herniation levels were comparable in the dropouts and in the patients who were investigated.

### Kinesiophobia

80 patients answered the Tampa scale for kinesiophobia (TSK). Approximately half of them (36/80) reported kinesiophobia scores of > 37 on the TSK (Vlaeyen et al. 1995b). Descriptive data were comparable between the groups with and without kinesiophobia regarding age, sex, place of birth, number of operations, and disc herniation level. Before surgery, patients with kinesiophobia had not experienced symptoms any longer than the patients without kinesiophobia (Table 1).

### Kinesiophobia in relation to outcome measures

Patients with kinesiophobia showed statistically significantly poorer results in 8 of 10 outcome measures in comparison to those without kinesiophobia (Table 2 and 3).

**Table 2.** Kinesiophobia vs. no kinesiophobia

Assessments	Kinesiophobia **^a^**		No kinesiophobia **^a^**		p-value
	median (range)	n	median (range)	n	
ODI **^b^**	32 (0–74)	36	12 (0–64)	43	< 0.001
EQ-5D**^index^^c^**	0.72 (–0.07 to 1.0)	35	0.80 (–0.16 to 1.0)	43	0.01
EQ-5D**^VAS^^d^**	68 (15–97)	36	80 (2–100)	44	0.01
VAS back pain **^e^**	44 (0–89)	36	12 (0–87)	44	< 0.001
VAS leg pain **^e^**	23 (0–91)	35	8 (0–84)	44	0.01
ZDS **^f^**	40 (22–67)	33	34 (22–51)	41	0.01
PCS **^g^**	26 (4–45)	33	14 (0–28)	40	0.01
SES **^h^**	35 (12–62)	34	51 (19–64)	41	< 0.001

**^a^** Kinesiophobia is defined as > 37 on the Tampa scale for kinesiophobia (TSK), which ranges from 17 to 68, with lower score indicating less severe symptoms.

**^b^** Oswestry disability index (ODI) ranges from 0 to 100, with lower score indicating less severe symptoms.

**^c^** European quality of life in 5 dimensions questionnaire (EQ-5D^index^) ranges from –0.59 to 1.0 where 1.0 is optimal health.

**^d^** EuroQol visual analog scale (EQ-5D^VAS^) ranges from 0 to 100, with higher score indicating less severe symptoms.

**^e^** Visual analog scales (VAS) for back and leg pain range from 0 to 100, with lower score indicating less severe symptoms.

**^f^** Zung self-rating depression scale (ZDS) ranges from 20 to 80, with lower score indicating less severe symptoms.

**^g^** Pain catastrophizing scale (PCS) ranges from 0 to 52, with lower score indicating less severe symptoms.

**^h^** Self-efficacy scale (SES) ranges from 8 to 64, with higher score indicating less severe symptoms.

**Table 3.** Work status and satisfaction with treatment

	Kinesiophobia		
	Yes	No	
	n = 36	n = 44	p-value
Work status			0.07
Working full-time/part-time	24	38	
Sick leave full-time	12	6	
Satisfaction with treatment			0.1
Satisfied with treatment	21	34	
Less satisfied/dissatisfied	15	10	

### Back-specific function

In ODI, 45 of 79 patients had minimal or no disability (0–20%). Of those, 14 reported kinesiophobia. 18 of the 79 patients had severe disability (> 40) in ODI, and 15 of the 18 reported kinesiophobia.

### Health-related quality of life

12 of 78 patients reported normal HRQoL (≥ 0.86 on EQ-5D), with 2 patients reporting kinesiophobia.

### Pain

39 of 79 patients had no leg pain. 14 of those 39 had kinesiophobia. 23 patients had no back pain, and 4 of them had kinesiophobia. 19 patients had neither leg pain nor back pain, but 4 of them had kinesiophobia.

### Depressive symptoms

45 of 74 patients who answered ZDS reported having symptoms of depression; 25 of them reported kinesiophobia.

### Catastrophizing thoughts

30 of 73 patients were non-catastrophizers, 10 of whom reported kinesiophobia. 21 of the 73 patients were catastrophizers, 18 of whom had kinesiophobia.

## Discussion

We found that half of the patients suffered from kinesiophobia 10–34 months after surgery for disc herniation. The patients with kinesiophobia were more disabled, had more pain, had more catastrophizing thoughts, had more symptoms of depression, had lower self-efficacy, and had worse HRQoL than patients without kinesiophobia.

After discectomy, leg pain is often reduced—as was found also in this study—but almost half of the patients still reported kinesiophobia. One possible explanation for this could be that fear of pain remains, even though the sensory component is gone. Pain-related fear develops if pain is interpreted as threatening (Vlaeyen et al. 1995a). Fear of pain is also linked to psychological factors such as kinesiophobia, depression, catastrophizing, and self-efficacy. The aim of surgery is primarily to relieve leg pain. Psychological factors are not attended by surgery. For example, both fear of movement and pain catastrophizing were found to be unaffected by behavior-graded activity in a randomized study 6 weeks after surgery (Ostelo et al. 2003a, b). Furthermore, it has been reported that intervention regarding psychological factors before surgery is beneficial for the outcome of surgery (Sullivan et al. 2009).

It was surprising that such a high degree of kinesiophobia was reported after surgery despite the patients being recommended exercise according to an early, active rehabilitation program (Kjellby-Wendt et al. 2001). A recent review showed that exercise programs after lumbar disc surgery lead to faster reduction of pain and disability than no such treatment (Ostelo et al. 2009). A Swedish study investigated effectiveness of 2 types of rehabilitation after lumbar fusion surgery: exercise therapy and psychomotor therapy (Abbott et al. 2010). In that study, the exercise therapy group had higher levels of kinesiophobia than the discectomy patients in our study, although they received similar early, active rehabilitation. This might be explained by the fact that the healing process after lumbar fusion takes longer than after discectomy. Moreover, it was surprising that the psychomotor therapy group in the study by Abbott et al. (2010) had better TSK values after lumbar fusion than the patients in our study did after discectomy. The psychological content of psychomotor therapy intervention would most likely explain these differences. The patients in the psychomotor therapy group also received a longer period of physiotherapy than the patients after disc surgery.

The TSK was developed to discriminate between non-excessive fear and phobia in patients with chronic musculoskeletal pain (Kori 1990). In the present study, we used TSK for patients after lumbar disc surgery. Often the patient with disc herniation has a background history that includes repeated periods of back problems, sometimes periods of leg pain and with increasing levels of intensity of pain. Before surgery, the level of pain is often very high. Patients with disc herniation may therefore be different from patients with chronic musculoskeletal pain, which TSK was developed for. Perhaps another cut-off value for kinesiophobia would be appropriate for patients with disc herniation.

Patients with kinesiophobia had had pain for the same period of time preoperatively as patients without kinesiophobia. Similarly, Börsbo et al. (2009) found that duration of pain appeared to have minor association with disability and quality of life in patients with chronic pain. Börsbo et al. (2009) investigated the interplay between intensity of pain, depression, anxiety, and catastrophizing but they did not include kinesiophobia.

Patients with a high degree of kinesiophobia also tend to have more depressive symptoms. It is well-known that pain and depression are closely linked. Another study found that more than half of the patients who reported anxiety/depression before surgery still felt the same after being operated (Jansson et al. 2005). Arpino et al. (2004) suggested that depression is an independent predictor of poor outcome after lumbar disc surgery. Since depression is known to be a consequence of kinesiophobia, according the original fear-avoidance model, preventing kinesiophobia may reduce depressive symptoms.

In summary, we found that patients who reported a high degree of kinesiophobia were also more affected in several other variables. We would argue that kinesiophobia is a factor that should be considered throughout the rehabilitation process.
